# Generation of Remosomes by the SWI/SNF Chromatin Remodeler Family

**DOI:** 10.1038/s41598-019-50572-8

**Published:** 2019-10-02

**Authors:** Manu Shubhdarshan Shukla, Sajad Hussain Syed, Ramachandran Boopathi, Elsa Ben Simon, Sunil Nahata, Lorrie Ramos, Defne Dalkara, Cendrine Moskalenko, Andrew Travers, Dimitar Angelov, Stefan Dimitrov, Ali Hamiche, Jan Bednar

**Affiliations:** 1 Université de Lyon, Laboratoire de Biologie et Modélisation de la Cellule, CNRS-UMR 5239, Ecole Normale Supérieure de Lyon, 46 Allée d’Italie, 69364 Lyon, cedex 07 France; 2 Université Grenoble Alpes, CNRS UMR 5309, INSERM U1209, Institute for Advanced Biosciences (IAB), Site Santé - Allée des Alpes, 38700 La Tronche, France; 3 Laboratoire de Physique, UMR 5672, CNRS, Université de Lyon 1, Ecole Normale Supérieure de Lyon, 69364 Lyon, cedex 07 France; 4 MRC Laboratory of Molecular Biology, Hills Road, Cambridge, CB2 2QH UK; 5 Izmir Biomedicine and Genome Center, Izmir, Turkey; 6 Institut de Génétique et de Biologie Moléculaire et Cellulaire, CNRS/INSERM/ULP, Parc d’innovation, 1 rue Laurent Fries, 67404 Ilkirch, Cedex France; 7 Cellule LBMC, 46 Allée d’Italie, 69007 Lyon, France; 8 Laboratory of the Biology and Pathology of the Eye, Institute of Biology and Medical Genetics and Institute of Medical Biochemistry and Laboratory Diagnostics, First Faculty of Medicine, Charles University and General University Hospital in Prague, Albertov 4, 128 00 Prague 2, Czech Republic; 9 Present Address: Wellcome Centre for Cell Biology and Institute of Cell Biology, School of Biological Sciences, The University of Edinburgh, Swann Building, King’s Buildings, Mayfield Road, Edinburgh, EH9 3BF United Kingdom; 10 Present Address: Pharmacology Division, CSIR-IIIM, Sanatnagar, Srinagar, 190005 Jammu and Kashmir India

**Keywords:** Chromatin remodelling, Atomic force microscopy

## Abstract

Chromatin remodelers are complexes able to both alter histone-DNA interactions and to mobilize nucleosomes. The mechanism of their action and the conformation of remodeled nucleosomes remain a matter of debates. In this work we compared the type and structure of the products of nucleosome remodeling by SWI/SNF and ACF complexes using high-resolution microscopy combined with novel biochemical approaches. We find that SWI/SNF generates a multitude of nucleosome-like metastable particles termed “remosomes”. Restriction enzyme accessibility assay, DNase I footprinting and AFM experiments reveal perturbed histone-DNA interactions within these particles. Electron cryo-microscopy shows that remosomes adopt a variety of different structures with variable irregular DNA path, similar to those described upon RSC remodeling. Remosome DNA accessibility to restriction enzymes is also markedly increased. We suggest that the generation of remosomes is a common feature of the SWI/SNF family remodelers. In contrast, the ACF remodeler, belonging to ISWI family, only produces repositioned nucleosomes and no evidence for particles associated with extra DNA, or perturbed DNA paths was found. The remosome generation by the SWI/SNF type of remodelers may represent a novel mechanism involved in processes where nucleosomal DNA accessibility is required, such as DNA repair or transcription regulation.

## Introduction

Chromatin has a repeating structure, whose underlying unit is the nucleosome, a nucleoprotein complex consisting of an octamer of core histones (two each of H2A, H2B, H3 and H4) and ∼150 bp of DNA, which is wrapped around the histone core in ∼1,65 left-handed turns^1^. The structure of both the histone octamer^2^ and the nucleosome core particle^3^ has been solved by X-ray crystallography. The individual histones exhibit a “histone-fold” structured domain and non-structured, highly flexible NH_2_-termini, which protrude from the nucleosome. The nucleosomes are connected by so-called linker DNA and a fifth histone, the linker histone, is associated with this DNA^1^. The globular domain of the linker histone is internally located in the 30 nm chromatin fiber^4^ and binds to entrance and exit nucleosomal DNA^5^. Nucleosomal arrays are further folded into the thick 30 nm chromatin fiber and this folding is assisted by the linker histones and the NH_2_-core histone termini^6–8^. The NH_2_-core histone termini interact with both the nucleosomal and the linker DNA^9^ and modifications of the tails are involved in the assembly of the mitotic chromosomes^10,11^.

Nucleosomes are stable particles and hence interfere with cellular processes requiring access to genomic DNA (reviewed in^12^). The cell uses three main strategies to overcome nucleosomal barriers and to gain access to nucleosomal DNA. These involve histone modifications (reviewed in^13^), histone variants (reviewed in^14^) and chromatin remodeling complexes (reviewed in^15,16^). Chromatin remodeling complexes are multiprotein assemblies containing variable numbers of subunits^15,17–19^. Each remodeling complex, however, contains an ATPase, which possesses DNA translocase properties and is essential for the function of the complex. According to the type of ATPase, the chromatin remodeling complexes can be divided into at least four distinct families: SWI2/SNF2, ISWI, CHD and INO80^16,20^. The complexes from the different groups exhibit a common property, namely, they can mobilize the histone octamer using energy freed from ATP hydrolysis^18,21^. In addition, the complexes from the SWI2/SNF2 family (SWI/SNF and RSC) induce strong perturbations of histone-DNA interactions and can evict the histone octamer from nucleosomal DNA^22–24^. It was also shown that Swr1, a complex belonging to the INO80 family, possesses novel properties and is required for the exchange of the histone variant H2A.Z^25^. Note that alterations in nucleosome structure, induced by the incorporation of some histone variants, also affect the capacity of chromatin remodelers to mobilize the resulting nucleosomes^26–28^.

SWI/SNF, the first chromatin remodeling complex discovered^29^, is involved in several processes, including transcription^29^, DNA repair^30^, splicing^31^ and telomeric and ribosomal DNA silencing^32^. It consists of roughly 11 subunits and has a central cavity. The dimensions of the cavity (diameter ∼15 nm, depth ∼5 nm) fit well with those of the nucleosome, suggesting that it may be a nucleosome-binding pocket^33,34^. This suggests that SWI/SNF would interact and remodel only one nucleosome at the time.

ACF belongs to the ISWI family of chromatin remodelers. The ISWI chromatin remodelers are involved in transcriptional regulation, in particular transcriptional repression, and chromatin assembly^35–37^. Human ACF, which contains two subunits, a catalytic subunit SNF2h and a non-catalytic one, ACF1, is able to generate *in vitro* arrays with evenly spaced nucleosomes^36,38^. The histone chaperone NAP-1 is found to be required for the assembly of the arrays^39^. Single molecule techniques suggest that ACF is able to assemble regularly spaced arrays with 50–60 bp linkers by kinetically discriminating against shorter linker DNAs^40^.

Despite numerous studies, the mechanism (or mechanisms) of action of the remodeling complexes is far from being clear. Two classes of mechanistic models have been proposed (for reviews see^16,20^). According to the first class, DNA moves on the surface of the histone octamer in 1 bp waves. This model is, however, inconsistent with several recent reports (for review see^20^). In the second class of models, the remodeler creates a bulge on the nucleosomal surface, which is then directionally propagated^20^. Since the dimensions of SWI/SNF are quite large and its contacts with DNA are extensive (the nucleosome is supposed to occupy the SWI/SNF cavity), a large segment of DNA could be involved in the bulge formation^41^. Note that both classes of models described the nucleosome mobilization as a continuous process, which is achieved without dissociation of the remodeler from the nucleosome. We have however recently reported that RSC remodeling is a two-step process, consisting of the initial formation of what we have termed as “remosome”, a non-mobilized particle with approximately 180 bp DNA loosely associated with the histone octamer. Nucleosome mobilization then occurs in a separate step^42^. This model is supported by experiments showing that the remosomes are intermediate products of the remodeling reaction, stable outside the remodeling complex, and acting as substrates for RSC leading to efficient mobilization^42^.

In this manuscript, we have studied the nucleosome mobilization mechanisms of two further remodelers, SWI/SNF, which belongs to the same family as RSC, and ACF, which belongs to the ISWI family. We demonstrate that these remodelers clearly use different mechanisms for nucleosome mobilization. SWI/SNF, in common with RSC, generates remosomes that are mobilized in a separate step, whereas ACF achieves mobilization without remosome formation.

## Results

### DNase I footprinting and restriction enzyme accessibility of the products of the SWI/SNF remodeling reaction

Using AFM, we have recently shown that the SWI/SNF nucleosome remodeling reaction mixture contains three types of particles: unperturbed nucleosomes (associated with ∼150 bp of DNA), mobilized nucleosomes, and nucleosome-like particles that have not been repositioned, but are associated with ∼175–180 bp of DNA^43^. These particles resemble the remosomes generated during the first step of the RSC nucleosome remodeling reaction (compare Fig. 4 of^43^ with Fig. 2 of^42^). We will consequently refer to these particles as SWI/SNF remosomes. Since they appear to be very similar to RSC remosomes, we expect them to involve similar perturbations of the DNA-histone interactions.

**Figure 1:**
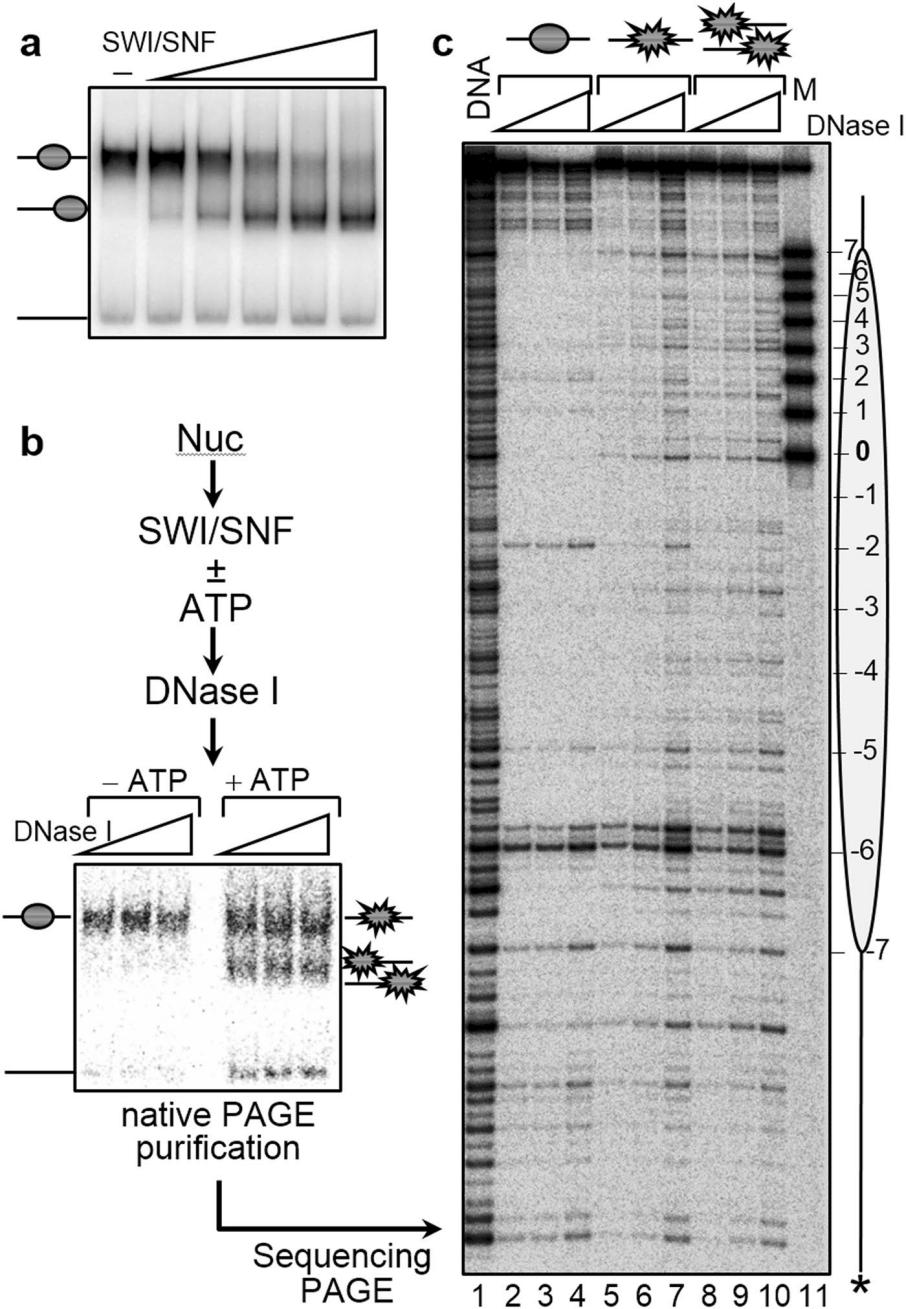
DNase I footprinting analysis shows that nucleosome treatment with SWI/SNF results in nucleosome remodeling prior to their mobilization. (**a**) Nucleosome mobilization with SWI/SNF. Centrally positioned nucleosomes on 601.1 DNA were incubated in presence of increasing amounts of SWI/SNF (as indicated) for 45 minutes at 29 °C. Reactions were stopped by the addition of 0.01 units of apyrase and the reaction products were resolved on a 5% native PAGE. Positions of free DNA, unremodeled and remodeled nucleosomes are indicated. (**b**) Schematics of the DNase I footprinting analysis of remosomes. Centrally positioned nucleosomes, reconstituted on a 255 bp 601.2 DNA sequence were incubated with SWI/SNF at 29 °C in the presence of ATP to generate ~50% repositioned particles (the error in the quantification does not exceed 5–7%). Then the reaction was stopped with apyrase and aliquots were incubated with increasing amounts of DNase I for 2.5 minutes at room temperature. After stopping the DNase I digestion reaction, the samples were separated on a 5% PAGE under native conditions. The bands corresponding to either the unremodeled particles (upper band) or remodeled particles (lower band) were excised from the gel, the DNase I digested DNA was eluted from the gel slices and run on an 8% sequencing gel. **(c)** DNase I footprinting. DNase I digestion pattern of control nucleosomes (lanes 1–3) and SWI/SNF treated nucleosomes isolated from the upper band (unremodeled particles, lanes 4–6) and the lower band (remodeled particles, lanes 7–9). The position of the histone octamer relative to the ends of the 601 DNA sequence and the nucleosome dyad are indicated on the left. Lane 10, DNase I digestion pattern of naked DNA.

To test this, we have used a combination of DNaseI footprinting and restriction enzyme accessibility assay of the SWI/SNF treated nucleosomes. DNaseI footprinting allows to shed light of nucleosome remodeling, while the restriction accessibility assay directly reflects the alterations in the histone-DNA interactions within the nucleosome. Briefly, we have reconstituted centrally positioned nucleosomes, using highly purified recombinant histones and 256 bp 601 DNA. Under the conditions used the efficiency of reconstitution was very high (essentially no free DNA was observed in the reconstituted samples) and the reconstituted particles exhibited a typical nucleosomal organization (Fig. 1a). The centrally positioned ^32^P-end labeled nucleosomes were incubated with increasing amounts of SWI/SNF at 29 °C in the presence of ATP, the reaction was stopped using apyrase and run on a 5% native PAGE. Conditions were found where ∼50% of the nucleosomes were repositioned (Fig. 1a) (under these conditions the remodeled nucleosomes are the dominant intermediate product of the remodeling reaction^42^). The nucleosomes were then incubated with SWI/SNF under the same conditions and, after stopping the reaction, were treated with increasing amount of DNase I. The digested particles were separated on the gel and the upper band (containing the non-mobilized particles) and the lower band (consisting of mobilized particles) were cut out. DNA was extracted from the gel slices and run on an 8% denaturing PAGE (Fig. 1b,c). The digestion pattern of both the mobilized and non-mobilized particles, in contrast to that of the control particles (incubated with SWI/SNF in the absence of ATP and gel-eluted after native PAGE), were similar and close to those of naked DNA (Fig. 1c, compare lanes 4–6 and lanes 7–9 with lane 10). These alterations in the DNaseI footprinting pattern are indicative for nucleosome remodeling in both the mobilized and non-mobilized nucleosomes.

**Figure 2:**
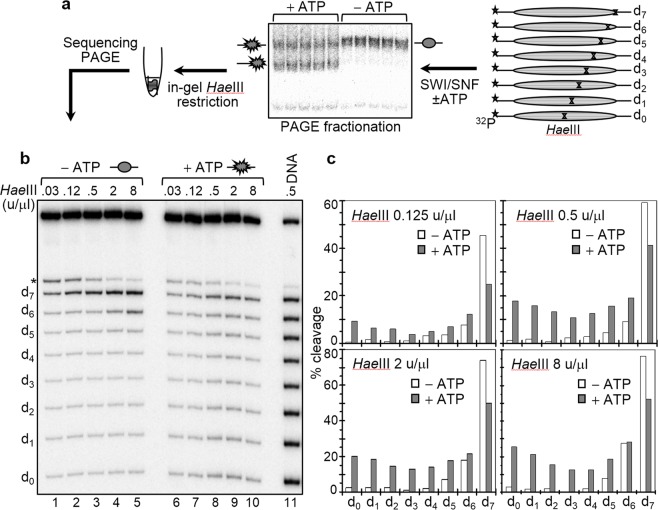
Measurements of the DNA accessibility towards *Hae*III along the length of nucleosomal DNA in control and SWI/SNF treated nucleosomes with the in gel one pot assay. (**a**) Schematics of the “in gel one pot assay”. **(b)** Electrophoresis under denaturing conditions of the *Hae*III digested DNA of the particles. Left panel, *Hae*III digestion pattern of control nucleosomes (incubated with SWI/SNF in the absence of ATP): right panel, same as left panel, but for nucleosomes treated with SWI/SNF in the presence of ATP although non-mobilized nucleosomes. After incubation with 2 units of SWI/SNF at 29 °C for 45 minutes and separation on a 5% native PAGE, the control and the non-mobilized nucleosome fractions were digested in the gel with the indicated amounts of *Hae*III for 5 minutes at 29 °C. The samples were then eluted from the gel slices, DNA was isolated and run on an 8% PAGE under denaturing conditions. Lane 11, in gel digested naked DNA with 0.5 u/μl of *Hae*III. (*) indicates a fragment which corresponds to an additional *Hae*III site present only in the d_7_ fragment 4 bp away from d_7_ itself. (**c**) Quantification of the data presented in (**b**).

To analyze the perturbations in the histone-DNA interactions within the SWI/SNF treated particles, we have used a recently developed approach, termed “in gel one pot assay” (see Fig. 2 and^42^). This approach allows the unambiguous detection of alterations in histone-DNA interactions with a 10 bp resolution along the totality of nucleosomal DNA. It is based on the restriction enzyme assay developed originally by Wu and Travers^44^. Briefly, eight mutated ^32^P-end labeled 255 bp 601.2 sequences were used to reconstitute centrally positioned nucleosomes (Fig. 2). Within each one of these sequences we introduced a single *Hae*III restriction site (designated as d_0_ to d_7_, where the subscript refers to the number of helical turns from the nucleosome dyad). Note that these restriction sites have identical rotational positions with outward-facing minor grooves^44^. The resulting nucleosomes were incubated with an appropriate amount of SWI/SNF (in the presence of ATP) to produce 50–60% of mobilized particles (judged by gel-shift, see Figure 2a) and the upper electrophoretic band, containing the remosome fraction, was excised and in gel digested with increasing amounts of *Hae*III. The digested DNA was purified from the gel and run on an 8% PAGE under denaturing conditions. A similar control experiment was also performed (incubating with SWI/SNF in the absence of ATP). The gel was then dried and the product bands were visualized by exposure on a Phosphorimager and quantified.

It can be seen (Fig. 2b,c), that the accessibility of the control particles to the restriction enzyme strongly decreases from d_7_ to d_0_. In fact, d_7_ and d_6_ behaved differently compared to the other positions since, even at the lowest concentration (0.125 u/µl) of *Hae*III, about 50% of d_7_ were accessible to the enzyme and this accessibility increases to 80% at the highest enzyme concentration (8 u/µl). In contrast, the internally located positions (from d_4_ to d_0_) were poorly cleaved at any concentration of *Hae*III. These results fully agree with the data of Wu and Travers^44^. However, upon nucleosome remodeling the *Hae*III accessibility changed dramatically at all positions (Fig. 2b,c). The accessibility of d_7_ decreased relative to the control particles, while that of the other positions strongly increased, with the largest change (10–12 fold increases in different experiments) observed at d_0_. We conclude that histone-DNA interactions within the SWI/SNF remosomes are strongly perturbed with the most significant modifications occurring in the vicinity of d_0_, close to the center of the particle. Note that, similar *Hae*III accessibility profiles were observed with RSC-generated remosomes^42^.

### Regions with *Hae*III accessibility similar to that of free DNA are present within the SWI/SNF remosomes

**Figure 3:**
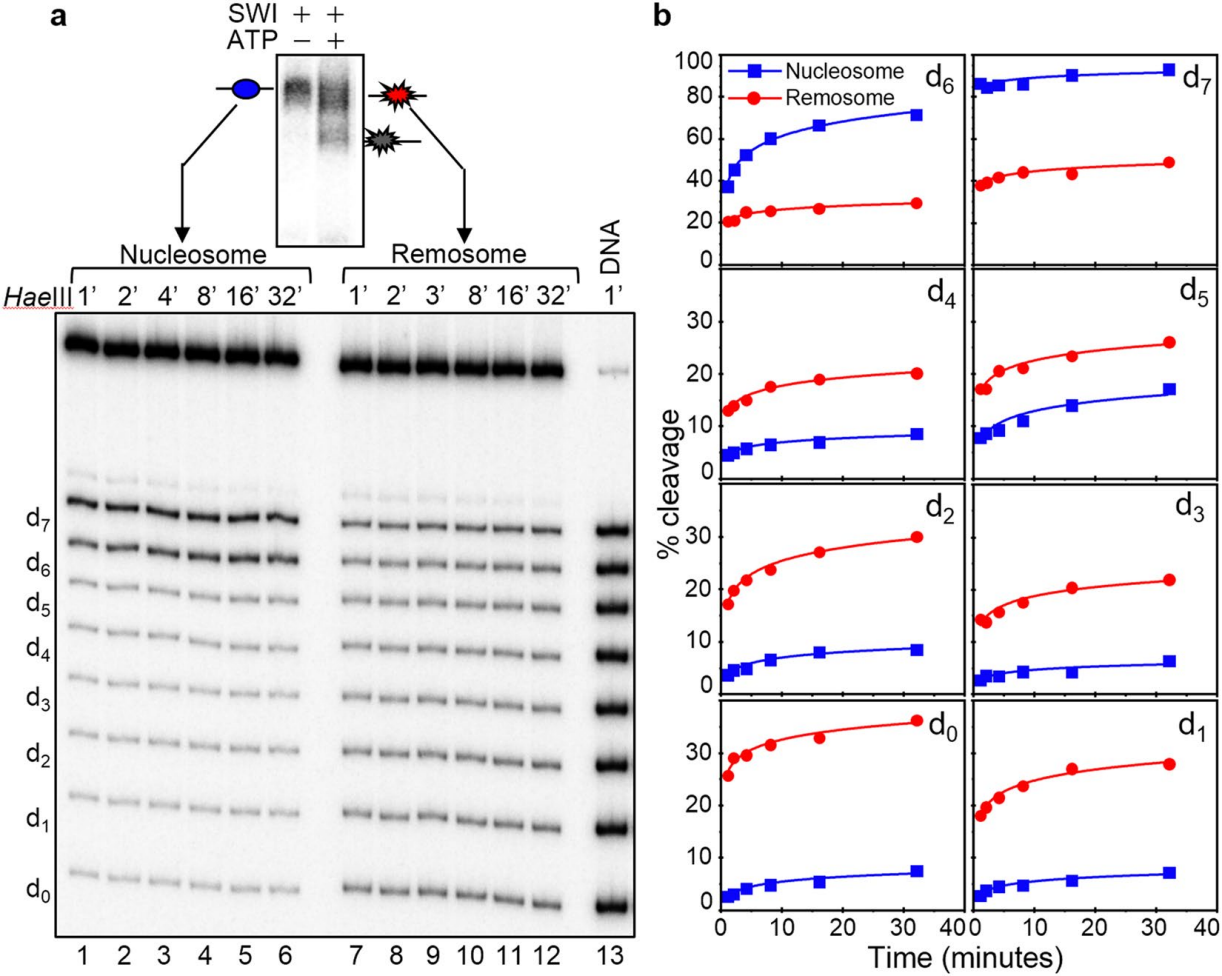
*Hae*III digestion kinetics of control nucleosomes and remosomes in solution. (**a**) Nucleosomes were reconstituted by using the eight ^32^P-labeled 255 bp 601.2 sequences, each containing a unique *Hae*III site (see Fig. 2a) and incubated with 2 units of SWI/SNF for 45 minutes at 29 °C. After running of the samples on a 5% PAGE, the control nucleosomes (incubated with SWI/SNF in the absence of ATP) and the SWI/SNF non-mobilized fraction were eluted from the gel in presence of unlabeled 601 nucleosomes. The same amount of both types of nucleosomes were digested with 2 u/μl of *Hae*III for different times, DNA from control nucleosomes (left panel lanes 1–6) and remosomes (right panel lanes 7–12) was isolated, purified and run on an 8% PAGE under denaturing conditions. The digestion times and the positions of the different restriction sites (d_n_) are indicated. Free DNA eluted in presence of same amount of unlabeled 601 nucleosomes was digested for 1 minute (Lane 13). **(b)** Quantification of the data presented in (**a**). Kinetic curves for *Hae*III accessibility are shown for non-remodeled nucleosomes (in blue) and remosomes (in red).

The “in gel one pot assay” shows that even at the lowest concentration of *Hae*III (0.125 u/μl) the *Hae*III accessibility of the internal positions (from d_0_ to d_4_) is quite high (for example, ∼10% for d_0_, see Fig. 2c), and up to 25% at the highest enzyme concentration (8 u/μl). Thus, there are DNA regions within the remosome that are easily cleaved by the enzyme and could well correspond to sites with highly perturbed histone-DNA interactions. To test this, we have gel purified the remosomes and the control nucleosomes and carried out *Hae*III digestion kinetics experiments in solution for both sets of particles (Fig. 3). For the control nucleosomes, the data show that the kinetics curves of *Hae*III accessibility at each position are smooth and monotonically increasing with time. However, for the remosomes, the time-dependent *Hae*III cleavage is quite different and the kinetics at each position show two well defined parts: an immediate cleavage (time point 1 min), and a relatively slow part comparable of that of control nucleosomes. The percentage of immediate cleavage is higher than that of the control nucleosomes at the same time point (Fig. 3b) with the exception of position d_7_. The greatest increase occurs at d_0_, where cleavage up to 10–15 fold higher than the control is observed. These data are in agreement with the “in gel one pot assay” results and further demonstrate that upon generation of remosomes the histone-DNA interactions are severely perturbed and free-DNA like regions are created. Similar behavior was exhibited by RSC remosomes upon digestion with *Hae*III^42^. Interestingly, it was recently found that another remodeler, INO80, was also able to generate intermediate particle with altered nucleosomal DNA accessibility, “reminiscent” to the remosome, suggesting INO80’s ability to generate an intermediate with altered DNA accessibility^45^. However, the observed enhanced restriction cutting in these particles, in contrast to the remosomes, was located only at 18 bp inside the nucleosome^45^.

### The DNA within SWI/SNF remosomes is strongly perturbed and follows variable paths around the histone core

**Figure 4:**
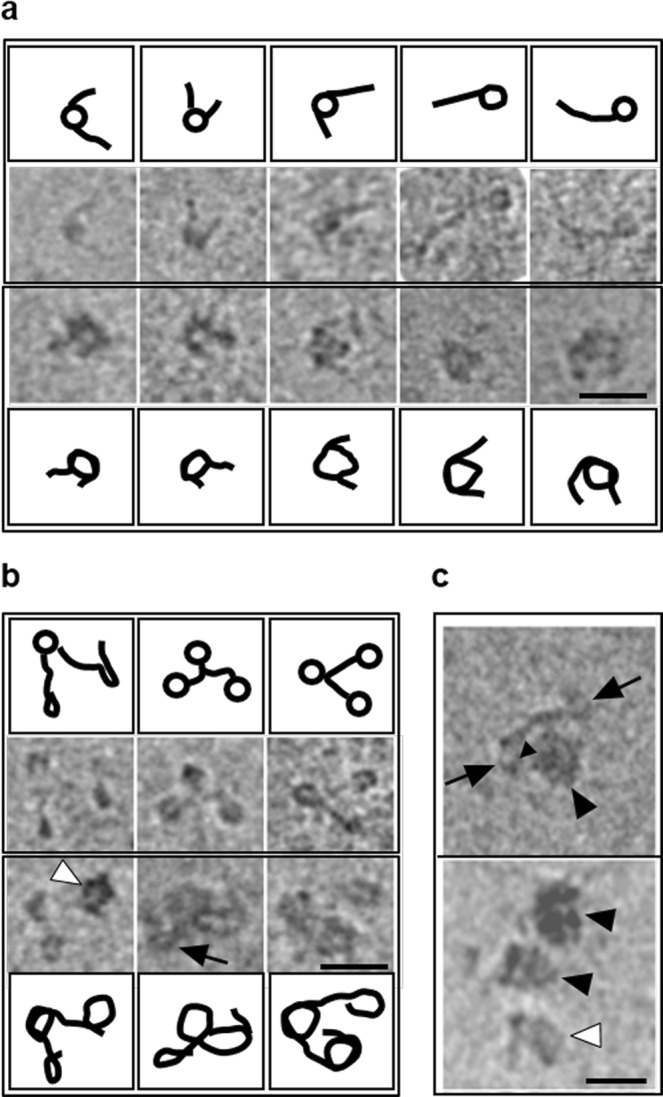
ECM visualization of remosomes. (**a**) Centrally positioned mononucleosomes were incubated in presence of SWI/SNF and ATP for 30 minutes at 29 °C (under these conditions ~40% of nucleosomes were mobilized to the end of the 601 DNA fragment). Upper panel shows nucleosomes that are either unperturbed (the first three micrographs) or repositioned (the last two micrographs) to the end of the DNA fragment by the action of SWI/SNF. The lower panel presents nucleosomes with altered structure. **(b)** SWI/SNF is able to alter nucleosomes within a trinucleosomal array. The trinucleosomal template was reconstituted on DNA fragment containing three repeats of the 601 sequence. The trinucleosomal array was remodeled in presence of SWI/SNF as in (**a**). The upper panel represents unaltered trinucleosomes, while the lower panel represents trinucleosomes altered by SWI/SNF. Note that all the nucleosomes can be altered by SWI/SNF (lower panel third column), only one nucleosome may remain unaltered (lower panel, second column, indicated by a black arrow), or only one nucleosome may be altered (lower panel, first column, indicated by a white arrow head). All the ECM micrographs are accompanied with line drawings illustrative of the shape of DNA observed in micrographs. **(c)** The SWI/SNF complex associates with a single nucleosome in a trinucleosomal array. SWI/SNF bound nucleosomes are indicated by black arrowheads. The black arrows indicate unaltered nucleosomes. The white arrow head indicates an altered, but unbound, nucleosome. Scale bar 50 nm.

The *Hae*III digestion pattern of the SWI/SNF remosomes could be interpreted in at least two ways: (i) as the result of a single, structurally deformed particle with varying DNA accessibility within the remosome or, (ii) as the result of a multitude of structures, each one exhibiting a distinct, altered DNA organization. To differentiate between these two possibilities, we have used electron cryo-microscopy (ECM) to obtain high-resolution images of the DNA within unfixed and unstained remosomes^27,42,46^. Briefly, we incubated centrally positioned nucleosomes with SWI/SNF in the presence of ATP (under conditions where ∼40% of nucleosome mobilization is achieved). An aliquot of the reaction mixture was then vitrified and used for ECM visualization. The cryo-electron micrographs (Fig. 4a) clearly show three types of structures: (i) a small amount of centrally positioned nucleosomes, which are indistinguishable from the control nucleosomes (Fig. 4a, upper panel, the first three micrographs); (ii) normally-shaped, but repositioned nucleosomes (Fig. 4a, upper panel, the last two micrographs); (iii) a multitude of different structures that we attribute to remosomes (Fig. 4a, lower panel). Typically, the structures in the latter category are larger and more irregular than the control nucleosome and are associated with shorter free DNA arms. We conclude that remosomes do not have single, well-defined structures, but rather can adopt a wide variety of distinct structures each with a different perturbed DNA organization. We recall that very similar structures were also observed with RSC-generated remosomes^42^.

We lastly studied SWI/SNF-generated remodeling products on trinucleosomes reconstituted on a DNA fragment containing three successive 601 repeats (Fig. 4b). Consistent with the data from mononucleosomes, SWI/SNF action on trinucleosomes again resulted in the generation of remosome-like particles characterized by shorter linker DNA and an increase in the particle diameters (Fig. 4b, compare the upper panel representing unremodeled trinucleosomes with the lower panel representing remodeled trinucleosomes). Interestingly, within one particular trinucleosome template, both remodeled and unremodeled nucleosomes were observed. We also observed a small fraction of trinucleosomes complexed with SWI/SNF (Fig. 4c). These particles are consistent with a single nucleosome being bound by a single SWI/SNF complex, following the dimensions reported in a previous study of this remodeler^33^. Taken together, these results support the idea that SWI/SNF remodels one nucleosome at a time.

### ACF nucleosome mobilization does not involve formation of remosomes

The data presented above and those described in^42^ show that SWI/SNF and RSC, remodelers belonging to the same family, both generate remosomes as part of their reaction mechanism. The question then arises whether remodelers from other families also act via remosome formation. We have addressed this question using ACF, a remodeler belonging to the ISWI family.

The main characteristic of remosomes is the presence of 30–40 bp of extra DNA loosely associated with the histone octamer with an accessibility close to that of free DNA (^42^ and this work). Does ACF remodeling lead to the formation of such particles?

**Figure 5:**
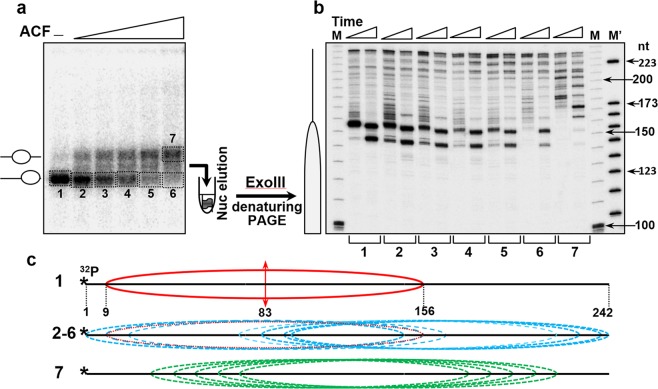
Exonuclease III mapping. (**a**) End-positioned nucleosomes were reconstituted on a^32^ P-labeled 242 bp 601 DNA sequence and incubated with increasing amounts of ACF. The reaction mixtures were then run on a 5% PAGE under native conditions, the bands corresponding to the higher mobility fractions (1–6) and to the lower mobility one (fraction 7) were excised from the gel and eluted. The gel eluted particles were next digested with 0.08 u/μl of Exo III for 1 and 2 minutes at 37 °C and the DNA was then purified (see schematics at the left part of the panel). **(b)** Determination of the nucleosome positions in the ACF remodeled particles. DNA, purified from the remodeled by ACF and Exo III cleaved particles was run on 8% PAGE under denaturing conditions. (M) and (M’), DNA size markers. The lengths of some of the markers (in nucleotides, nt) are noted on the right part of the figure. Left, schematic presentation of the position of the control, end-positioned nucleosome. **(c)** Schematics of the positions (relative to the DNA ends) of the control particles, not treated with ACF (1), the ACF remodeled particles with no changes in their electrophoretic mobility (fractions 2–6), and the particles with lower mobility (7). The single position of the control particle is shown in red, the positions of the mobilized particles from fractions 2–6 are in blue, and those within fraction 7 are in green.

We have reconstituted end-positioned nucleosomes using a 242 bp 601 DNA sequence (see schematics in Fig. 5c). We incubated these nucleosomes with increasing amounts of ACF at 29 °C in the presence of ATP and then ran the reaction products on a 5% native PAGE (Fig. 5a). In addition to a band with higher electrophoretic mobility, incubation with ACF results in the appearance of a band with lower mobility that corresponds to nucleosomes that have been repositioned towards the center of the DNA construct. As expected, increasing the amount of ACF in the remodeling reaction is paralleled by an increase in the intensity of the lower mobility band (Fig. 5a). To map the positions of the nucleosomes in each band, we have eluted the particles from the corresponding gel slices (slices 1–6 correspond to the nucleosome fractions co-migrating with the non-remodeled nucleosomes, while slice 7 corresponds to the repositioned nucleosomes, Fig. 5a) and digested them with Exo III (Fig. 5b).

Exo III cleavage of the control nucleosomes, untreated with ACF (slice 1), revealed a very strong band, reflecting strongly positioned nucleosomes at 156 bp from the 5′-end of the DNA (Fig. 5b, 1, first lane; see also schematics in Fig. 5c). Digestion with higher amounts of Exo III led to the appearance of second band at ∼145 bp from the 5′-end that originates from exonuclease arrest at roughly 10 bp from the nucleosome end due to very strong histone-DNA interactions at this site. In addition to these two bands, the cleavage pattern of the nucleosomes eluted from slices 2–6 showed a series of new bands in the upper part of the gel. The highest molecular mass band corresponds to a fragment of roughly 240 bp (Fig. 5c, 2–6). These bands were separated by 10 bp intervals and are attributed to the generation of ACF repositioned nucleosomes at the 3′-end of the DNA.

**Figure 6:**
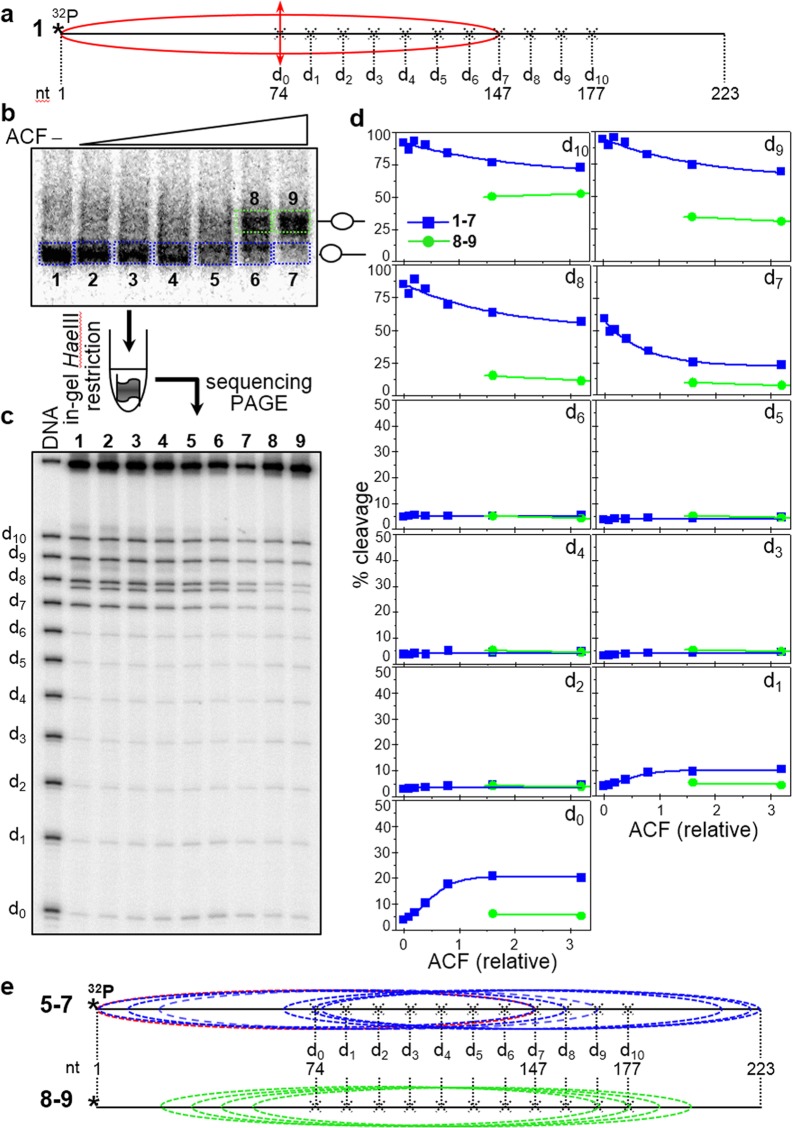
Time course of *Hae*III acessibility of ACF-remodeled nucleosomes in solution. (**a**) Schematics of the reconstituted nucleosomes. Eleven different ^32^P-end-labeled mutated 223 bp 601.2 DNA sequences were used to reconstitute end-positioned nucleosomes (each one of the sequences bears a unique *Hae*III restriction site, designated d_0_ to d_10_, where the number indicates their position in helical turns from the dyad). (see schematics presented in (**a**) and in Fig. 2a). (**b**) An equimolar mixture of the 11 end-positioned nucleosomes was incubated with increasing amounts of ACF in the presence of ATP. The reaction mixtures were then separated on a 5% PAGE under native conditions. Then the lower bands (containing the particles with non-modified mobility) as well as the upper bands (containing the particles with modified, lower mobility) were excised from the gel and the particles were eluted from the gel slices. The eluted samples were digested with 4 units/μl of *Hae*III for 5 minutes. The digested DNA was run on an 8% PAGE under denaturing conditions **(c)**. **(d)** Quantification of the data presented in (**c**). After exposure of the dried gel, *Hae*III digestion product bands for each restriction site the respective particle were quantified and expressed as a percentage of cut fraction in function of the amount of ACF used for remodeling; blues, fractions 1–7; green, fractions 8–9. **(e)** Upper panel, schematics of the Exo III mapped positions (relative to the DNA ends) of the nucleosomes within fractions 1–7 which exhibit non-modified (high) electrophoretic mobility; the position of the control particle, not treated with ACF is shown in red. Lower panel, same as the upper panel, but for the Exo III identified positions of the nucleosomes from fractions 8 and 9, showing modified, lower electrophoretic mobility.

**Figure 7:**
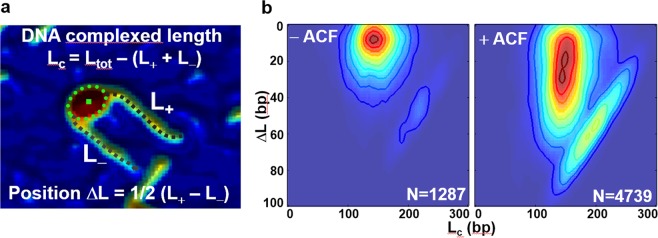
The ACF-remodeled nucleosomes are associated with ~150 bp of DNA. (**a**) Enlarged view of an AFM topography image of a centrally positioned nucleosome and schematics of the analysis. **(b)** Two-dimensional histogram L_C_/ΔL representing the complexed length of DNA L_C_ versus the nucleosome position ΔL. Left panel: nucleosomes incubated for 45 minutes at 30 °C in the absence of ACF (N = 1287 particles analyzed). Right panel: nucleosomes incubated with ACF (N = 4739 particles analyzed).

The positions detected for these nucleosomes are presented schematically in Fig. 5c, 2–6. As for the particles eluted from slice 7 (the lower mobility band), Exo III mapping showed nucleosomes positioned at 10 bp intervals on DNA. Note that the majority of the nucleosomes were positioned “inside” the DNA construct, leaving ∼20 bp DNA ends essentially free of nucleosomes (Fig. 5b, 7 and schematics, Fig. 5c, 7). In summary, Exo III mapping showed that the higher mobility band produced by ACF remodeling contains nucleosome located towards the DNA ends, separated by 10 bp intervals, while the lower mobility band contains nucleosomes repositioned towards the interior of the DNA fragment (Fig. 5c). In agreement, the DNAseI footprinting pattern of both higher mobility and low mobility particles, showed the appearance of new bands (see Supplementary Fig. S1) that reflects enhanced DNase I accessibility all along DNA and thus, nucleosome repositioning.

After characterizing the positioning of the ACF remodeling products, we next used the “in gel one pot assay” to determine whether the different ACF remodeled particles exhibit regions with high accessibility to *Hae*III (a characteristic feature of the remosomes). Briefly, eleven end-positioned nucleosomes containing a unique *Hae*III site (at positions termed d_0_ to d_10_ as above) were reconstituted (see schematics in Fig. 6a) using a 223 bp fragment containing the 601 DNA sequence. An equimolar mixture of these nucleosomes was incubated with increasing amounts of ACF, the low mobility bands (1–7) and the higher mobility bands (8, 9) were excised and in gel digested with 4 units/µl of *Hae*III at 29 °C for 5 minutes (Fig. 6b). The digested DNA was run on an 8% denaturing PAGE (Fig. 6c) and the percentage cleavage efficiency of *Hae*III was quantified (Fig. 6d). We find that the accessibility towards *Hae*III of positions d_1_-d_6_ for the higher mobility bands 1–7 is very low (less than 5% cleavage) and essentially does not change with increasing ACF concentration, while that for d_7_-d_10_ it is very high at low ACF concentration and decreases with increased ACF concentration, the largest decrease being observed at d_7_. The accessibility of d_0_ to *Hae*III is very low (∼5% cleavage) at low ACF concentration and some increase (up to 15–20%) is observed for the highest concentration of ACF used.

These accessibility results are totally different from those obtained with remosomes (see Figs 2 and 3) and can easily be explained based on the Exo III mapping of the nucleosome positions in the different gel mobility bands (see Fig. 5) without requiring any ACF-induced alterations in the structure of the nucleosomes. Schematics of the presumed positions of the nucleosomes in bands 1–7 are presented on Fig. 6e, 1–7. This shows that positions d_1_-d_6_ are internally located in all populations of ACF repositioned nucleosomes and they are therefore inaccessible to *Hae*III (c.f. Figs 3 and 4), while positions d_7_-d_10_ are internally located only in some populations (with d_7_ being the least exposed) and thus partially accessible to *Hae*III. As for the increased *Hae*III accessibility at d_0_, it reflects its closer localization towards the nucleosomal ends in the ACF-repositioned nucleosomes (see schematics Fig. 6e, 1–7). The *Hae*III accessibility of the positions in bands 8 and 9 can be also explained by the nucleosome positions following reaction with ACF. Indeed, d_9_ and d_10_ are located towards the ends of the repositioned nucleosomes, while all the other positions are internally located (see Fig. 6, schematics 8–9). We can conclude that no *Hae*III high accessibility regions are present within the ACF remodeling particles and, therefore, this data does not support the generation of remosomes by ACF.

### The histone octamer of ACF remodeled nucleosomes is associated with ∼150 bp of DNA

If ACF were to generate remosome-like particles, the intermediate product(s) of the remodeling reaction, should contain 30–40 bp of extra DNA, leading to histone octamers associated with 180–190 bp of DNA (^42^ and this work).

We have used AFM to search for such particles. AFM allows precise measurement of the length of DNA associated with the histone octamer and the position of the histone octamer relative to the DNA ends for single nucleosomes^28,42^. We have analyzed thousands of AFM images for both control and ACF-treated nucleosomes using specially developed image-processing software (see Materials & Methods section for details). Note that our AFM images showed very few nucleosomes associated with ACF (we however worked under sub-saturation conditions, with at most one ACF complex per 10–20 nucleosomes).

The data are presented as 2D ΔL/L_C_ maps, where ΔL is the position of the nucleosome relative to the free ends of DNA and L_C_ is the length of DNA complexed with the histone octamer (Fig. 7a). In these AFM studies, the length of DNA used for reconstitution of centrally positioned nucleosomes was 311 bp^28,42^. For the 2D ΔL/L_C_ maps, L_C_ was calculated as L_C_ = L_t_ − L_+_ − L_−_, where L_t_ is the total length of the 601-containing DNA used for reconstitution, and ΔL/2 = (L_+_ − L_−_)/2. The results show that the control nucleosomes in the 2D maps were characterized by L_C_ ∼150 bp, a result in good agreement with the biochemical characterization (Fig. 7b-left panel). The ACF-treated particles led to completely different 2D maps (Fig. 7b-right panel). The different particles measured had a constant L_C_ of ∼150 bp and values of ΔL/2 varying in the range ∼0–60 bp. These results demonstrate that: (i) no particles with more than ∼150 bp DNA associated with the histone octamer are present in the ACF remodeling reaction mixture and, (ii) ACF is able to mobilize a centrally-positioned nucleosome to within roughly 20 bp from the DNA ends. These results are in agreement with the biochemical experiments (Figs 5 and 6) and rule out remosome generation during ACF remodeling.

## Discussion

In this work we have made a detailed comparison of the type and structure of the products of nucleosome remodeling by SWI/SNF and ACF using high-resolution microscopy methods combined with novel biochemical approaches.

We find that, in addition to mobilized nucleosomes, SWI/SNF generates a multitude of nucleosome-like particles that we have termed remosomes. Our earlier AFM data showed that these particles are associated with ∼180 bp of DNA, instead of 147 bp as in standard nucleosomes^43^. The “in gel one pot assay” shows that the histone-DNA interactions within remosomes are markedly perturbed. The DNase I footprinting pattern of the remosomes is clearly different from that of standard nucleosomes and is closer to that of free DNA. Electron cryo-microscopy visualization of the remosomes shows that they are larger than standard nucleosomes and also that they adopt a variety of different structures. Each remosome has an irregular DNA path, which varies from one remosome to the next. The presence of *Hae*III immediate cleavage regions within the remosomal DNA is in accord with major DNA path perturbations occurring at various positions around the histone core. Recently, we have reported that the RSC induced mobilization of nucleosomes requires the generation of remosomes^42^. These remosomes exhibited very similar properties to those found with SWI/SNF^42^. We thus conclude that remosome generation appears to be a common feature of the SWI/SNF family of remodelers. However, this conclusion does not necessarily apply to other families. Indeed, we have shown here that the ACF remodeler, a member of the ISWI family, only leads to nucleosome repositioning and no evidence was found for particles associated with extra DNA, or with perturbed DNA paths.

We finally remark that the generation of remosomes by the SWI/SNF family could be important well beyond the process of chromatin remodeling. If remosomes can be generated with relatively little energy and can remain stable outside remodeling complexes, then they would affect chromatin structure and local DNA accessibility. This in turn could have a significant impact on a variety of processes within the cell nucleus including DNA repair, transcription and recombination.

## Materials and Methods

### Note

For all figures containing images of gels the original full size untreated gel images are presented in the Supplementary Fig. S2.

### DNA fragments

The 255, 311 and 242 bp 601.1 fragments used for reconstitution of centrally and end positioned nucleosomes respectively, were PCR amplified from pGEM-3Z-601 plasmid (Kindly provided by Dr. J. Widom and Dr. B. Bartholomew). DNA probes were 5′ labeled by using ^32^P labeled primers in the PCR reactions. DNA fragments for performing ‘One pot assay’ were PCR amplified from a set of mutant 601.2 sequences, each containing a unique *Hae*III site at different superhelical locations. For SWI/SNF assays, a 281 bp DNA fragment was PCR amplified and digested with *Sph*I to yield a 255 bp fragment with 57 and 51 bp linkers respectively (described in^42^). For ‘One pot assays’ on ACF remodeled nucleosomes, a 223 bp fragment was PCR amplified which positions histone octamer at one end of the DNA. The trinucleosome DNA for electron cryo-microscopy was purified as described earlier^47^.

### Proteins and nucleosome reconstitutions

Recombinant *Xenopus laevis* full length histones were expressed and purified from *E. coli* strain BL21(DE3) as described^48^. *S. cerevisae* SWI/SNF complex was purified by standard methods. Expression and purification of ACF was performed as described previously^49^. Nucleosome reconstitutions were performed by salt dialysis method as described in^50^. For experiments with radiolabeled DNA substrates 100 ng of ^32^P end labeled DNAs were mixed with 2.4 µg of unlabeled low affinity chicken erythrocyte DNA and used for reconstitution.

### Nucleosome remodeling reactions

Remodeling reactions were performed with 150 fmol of nucleosomes in remodeling buffer (RB) 10 mM Tris pH 7.4, 5% glycerol, 1 mM rATP, 2.5 mM MgCl_2_, 1 mM DTT, 100 μg/ml BSA, 50 mM NaCl, 0.01% NP40) in a volume of 7.5 μl at 29 °C. For sake of convenience, SWI/SNF amounts are expressed in units. The SWI/SNF and ACF units were defined as described before^51^. However, under the experimental conditions described nucleosomes were always in 10–15 molar excess with respect to SWI/SNF or ACF concentration even under the highest concentration of remodelers used.

### DNase I footprinting assay

The remodeling reaction was performed in remodeling buffer in a volume of 7.5 µl at 29 °C for 50 min. The control reactions did not receive ATP. 450 fmol (control reactions) or 900 fmol (remodeling reactions) of nucleosomes reconstituted on ^32^P- end labelled 255 bp 601.2 DNA were incubated with the amount of SWI/SNF sufficient to mobilize ~50% of the nucleosomes. Reactions were stopped by addition of 0.03 units of Apyrase and 3 µg of plasmid DNA. Reaction products were divided into three equal aliquots and increasing amounts of DNase I (0.6, 0.12, 0.25 units for control nucleosomes; 0.12, 0.25 and 0.5 units for remodeled nucleosomes respectively) were added to remodeled or control nucleosomes. EDTA was added to 20 mM to stop the DNase I cleavage. Unmobilized and mobilized fractions were resolved on native PAGE (29:1) in 0.25X TBE. Bands, corresponding to unremodeled, remodeled-unmobilized and mobilized nucleosomes were excised from the gel, DNA was eluted, filtered, deproteinized through phenol:chloroform treatment, precipitated and run on 8% denaturing PAGE.

### One pot Restriction enzyme assays

In gel one pot assays were performed essentially as described in^42^. For one pot restriction enzyme assay on gel eluted nucleosomes, centrally positioned 150 fmol or 300 fmol of 601.2 nucleosomes (for unremodeled and remodeled experimental sets respectively) were incubated with SWI/SNF in the remodeling reaction as described above. Reaction products were separated on a 5% native polyacrylamide gel. Bands corresponding to unmobilized fractions (unremodeled as well as remodeled) were excised and nucleosome particles were eluted in 80 μl elution buffer containing Tris 10 mM pH7.4, 0.25 mM EDTA and 10 mM NaCl, at 4 °C for 3 hours with gentle shaking. Elution buffer contained ~50 nM of cold 255 bp 601.1 nucleosomes for the stability of eluted nucleosomes. Eluted nucleosomes were filtered, concentrated using 100 kDa cutoff spin filters and adjusted to buffer restriction digestion conditions (10 mM Tris pH 7.6, 10 mM MgCl_2_, 50 mM NaCl, 1 mM DTT and 100 μg/ml BSA). *Hae*III was added to 2 u/µl and the reaction was allowed to proceed at 29 °C. At indicated time points, aliquots were taken and the reaction was stopped by addition of 0.1% SDS and 20 mM EDTA. DNA was extracted through phenol:chloroform, precipitated and run on 8% denaturing PAGE. Gels were dried, autoradiographed, scanned on phosphorimager and quantified using Multi Gauge software (Fuji).

### Exonuclease mapping

In order to map the nucleosome positioning of the ACF treated nucleosomes we titrated the 5′ labeled 242 bp end positioned 601 nucleosomes with increasing amounts of ACF. The reaction products were run in a 5% native PAGE. Fast migrating band corresponding to control nucleosome and ACF treated particles (control like fast migrating and slow migrating bands) were excised from the gel and soaked in 100 μl of buffer containing 10 mM Tris pH 7.6, 10 mM NaCl, 0.25 mM EDTA, 100 μg/ml BSA and 3.5 μg/ml cold reconstituted nucleosomes. The samples were let to elute on shaker for 3 hours at 4 °C. Eluted samples were filtered and concentrated to 40 μl. MgCl_2_ and Exonuclease III were then added to a final concentration of 2.5 mM and 80 u/ml respectively. Aliquots were taken and reaction stopped at 1 and 2 minutes by stop buffer (0.1% SDS, 25 mM EDTA). DNA from the samples was extracted by phenol chloroform ethanol precipitation and run on an 8% denaturing gel.

### High-resolution Microscopy

For the AFM imaging, the nucleosomes were immobilized onto APTES-mica surfaces, imaged and analyzed as described previously^43^. DNA complexed length (L_C_) and position (ΔL) distributions were constructed as described in^42^. Nucleosomes samples for electron cryo-microscopy were prepared as described^42,52^.

## Supplementary information

Supplementary information

## Data Availability

No datasets were generated or analyzed during the current study.
