# Supplementary material for: Generation of Remosomes by the SWI/SNF Chromatin Remodeler Family
Source: Sci Rep. 2019 Oct 2;9:14212. doi: 10.1038/s41598-019-50572-8 (PMC6775096; doi:10.1038/s41598-019-50572-8)
Supplement: Supplementary file 1 — Supplementary information [file 41598_2019_50572_MOESM1_ESM.docx]

**Generation of Remosomes by the SWI/SNF Chromatin Remodeler Family**

Manu Shubhdarshan Shukla^1,2¶,#,*^, Sajad Hussain Syed^1,2¶,+^, Ramachandran Boopathi^1,2^, Elsa Ben Simon^1^, Sunil Nahata^1,2^, Lorrie Ramos^2^, Defne Dalkara^2^, Cendrine Moskalenko^3^, Andrew Travers^4^, Dimitar Angelov^1^, Stefan Dimitrov^2,5^, Ali Hamiche^6*^, and Jan Bednar^2,7,8*^

^1^Université de Lyon, Laboratoire de Biologie et Modélisation de la Cellule , CNRS-UMR 5239, Ecole Normale Supérieure de Lyon, 46 Allée d'Italie, 69364 Lyon cedex 07, France

^2^Université Grenoble Alpes, CNRS UMR 5309, INSERM U1209, Institute for Advanced Biosciences (IAB), Site Santé - Allée des Alpes, 38700, La Tronche, France

^3^Laboratoire de Physique, UMR 5672, CNRS, Université de Lyon 1, Ecole Normale Supérieure de Lyon, 69364 Lyon cedex 07, France

^4^MRC Laboratory of Molecular Biology, Hills Road, Cambridge CB2 2QH, UK

^5^"Roumen Tsanev" Institute of Molecular Biology, Bulgarian Academy of Sciences, Sofia, Bulgaria

^6^Institut de Génétique et de Biologie Moléculaire et Cellulaire, CNRS/INSERM/ULP, Parc d’innovation, 1 rue Laurent Fries, 67404 Ilkirch Cedex, France

Cellule LBMC, 46 Allée d'Italie, 69007, Lyon, France

^7^Laboratory of the Biology and Pathology of the Eye, Institute of Biology and Medical Genetics, First Faculty of Medicine, Charles University and General University Hospital in Prague, Albertov 4, 128 00 Prague 2, Czech Republic

^¶^These authors contributed equally

^#^Present address: Wellcome Centre for Cell Biology and Institute of Cell Biology, School of Biological Sciences, The University of Edinburgh, Swann Building, King’s Buildings, Mayfield Road, Edinburgh EH9 3BF, United Kingdom

^+^Present address: Pharmacology Division, CSIR-IIIM, Sanatnagar, Srinagar-190005, Jammu and Kashmir, India

*Correspondence: [v1mshukl@exseed.ed.ac.uk](mailto:v1mshukl@exseed.ed.ac.uk) (M.S.), hamiche@igbmc.fr (A.H.), [jan.bednar@univ-grenoble-alpes.fr](mailto:jan.bednar@univ-grenoble-alpes.fr) (J.B.)

^8^Lead Contact: jan.bednar@univ-grenoble-alpes.fr

**Supplementary material :**

**Figure S1. DNase I footprinting analysis of ACF treated nucleosomes.**


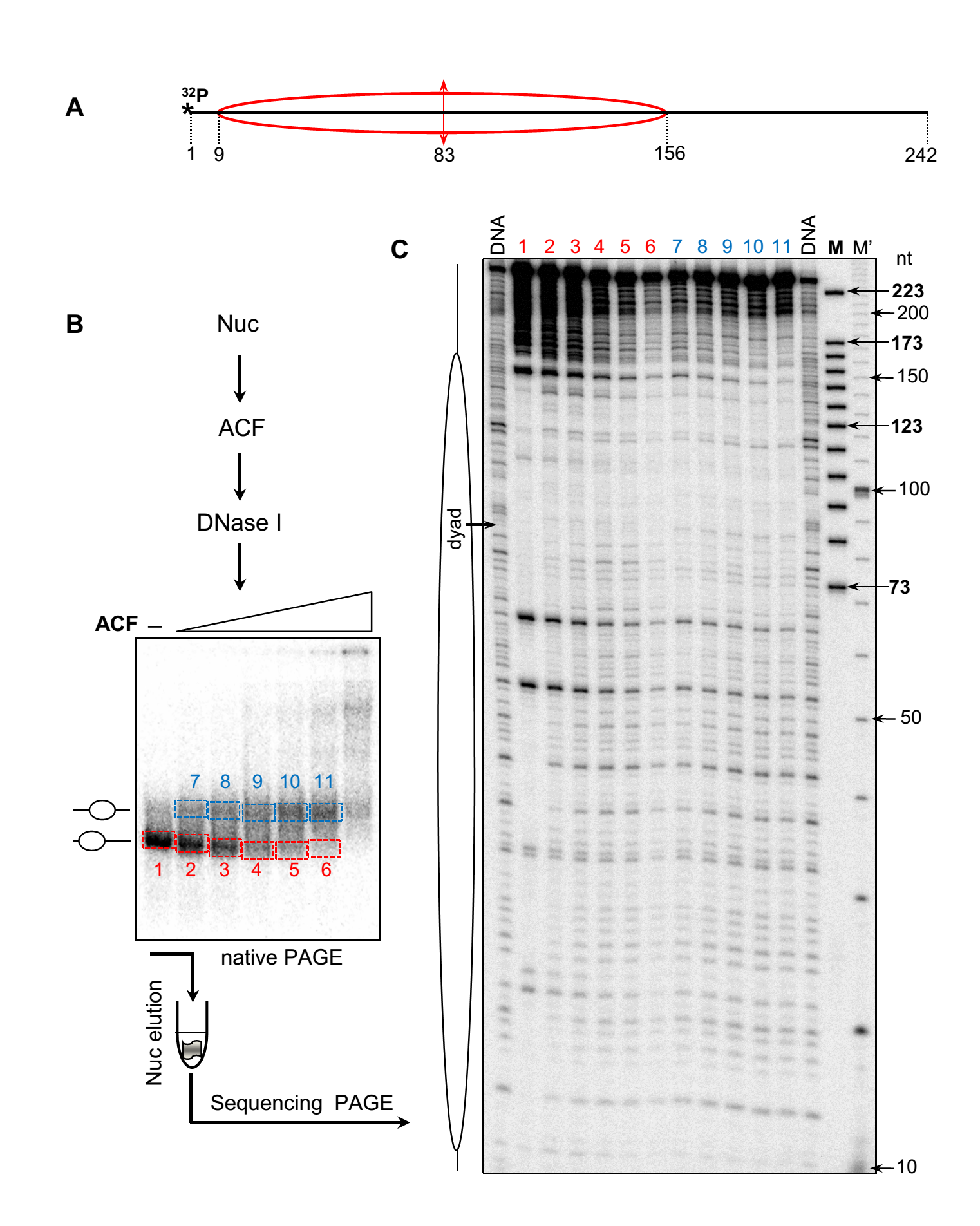


**(A)** Schematics showing the reconstituted 601-end positioned nucleosome used in the ACF experiments. The nucleosomes were ^32^P-end labeled. **(B)** Schematics of the DNase I footprinting analysis of the ACF-treated nucleosomes. End-positioned nucleosomes were incubated with increasing amount of ACF at 29 °C in the presence of ATP. Then the reaction was stopped with apyrase and aliquots were incubated with increasing amounts of DNase I for 2.5 minutes at room temperature. After stopping the DNase I digestion reaction, the samples were separated on a 5% PAGE under native conditions. The bands corresponding to either the slid particles (upper band) or unshifted particles (lower band) were excised from the gel, the DNase I digested DNA was eluted from the gel slices and run on an 8% sequencing gel. **(C)** DNase I footprinting. DNase I digestion pattern of control nucleosomes (lanes 1) and ACF-treated nucleosomes isolated from either the lower band (unshifted particles, lanes 2-6) or the upper band (slid particles, lanes 7-9). The position of the histone octamer relative to the ends of the 601 DNA sequence and the nucleosome dyad are indicated on the left. DNA, DNase I digestion pattern of naked DNA.

**Figure S2** : Images of original gels used for the assembly of the figures in the main text. The numbers denote corresponding numbers and parts of the main text figures and the parts of the gels used for the figure assembly are deimited by the black rectangles.







1A





1B

1C







2A

2B


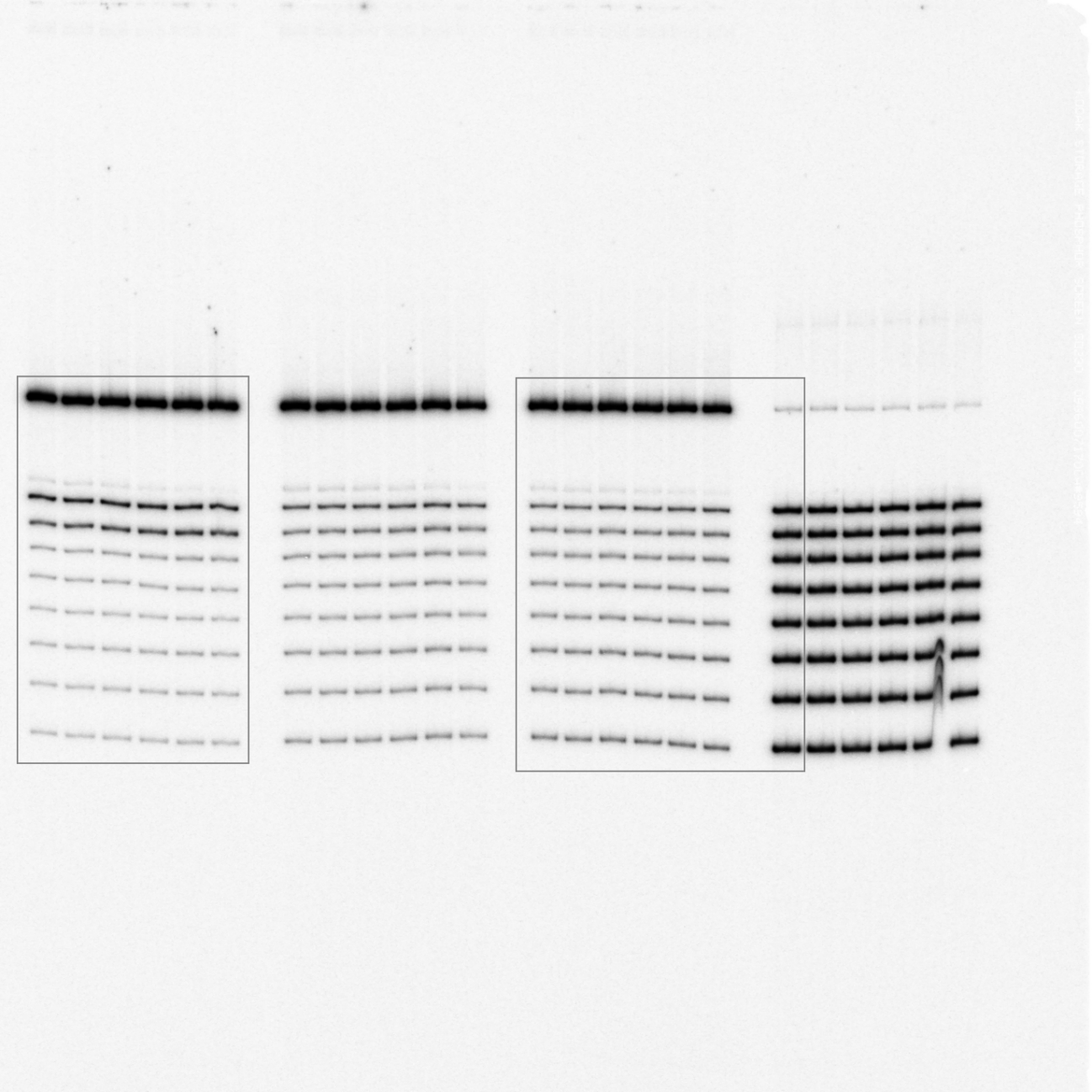


3A







5A

5B








6A

6B


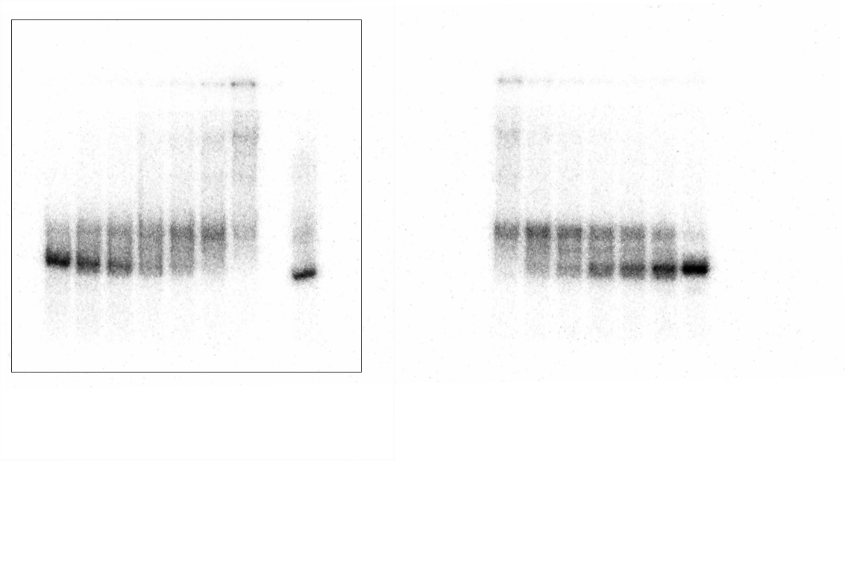
 S1B





S1C
